# Risk Factor and Surgical Outcome of Petersen’s Hernia After Gastrectomy in Gastric Cancer

**DOI:** 10.3389/fonc.2021.765509

**Published:** 2021-11-08

**Authors:** Song Liu, Qiongyuan Hu, Peng Song, Liang Tao, Shichao Ai, Ji Miao, Feng Wang, Xing Kang, Xiaofei Shen, Feng Sun, Xuefeng Xia, Meng Wang, Xiaofeng Lu, Wenxian Guan

**Affiliations:** ^1^ Department of Gastrointestinal Surgery, Nanjing Drum Tower Hospital, The Affiliated Hospital of Nanjing University Medical School, Nanjing, China; ^2^ Department of Gastrointestinal Surgery, Nanjing Drum Tower Hospital, Drum Tower Clinical Medical College of Nanjing Medical University, Nanjing, China

**Keywords:** internal hernia, gastrectomy, gastric cancer, risk factor, Petersen’s hernia

## Abstract

**Background:**

Petersen’s hernia is a life-threatening complication after gastrectomy. This study is dedicated to identify risk factors for Petersen’s hernia and compare clinical outcomes between patients receiving early or delayed surgical interventions.

**Methods:**

Data from all patients who received gastrectomy due to gastric cancer were collected. Clinical characteristics were compared between Petersen and non-Petersen groups, bowel necrosis and non-necrotic groups. Propensity score matching (PSM) was conducted to generate two comparative groups. Univariate analysis and multivariate logistic regression were performed for risk factor evaluation.

**Results:**

A total of 24 cases of Petersen’s hernia were identified from 1,481 cases of gastrectomy. PSM demonstrated that lower body mass index [BMI; odds ratio (OR) = 0.2, p < 0.01] and distal gastrectomy (OR = 6.2, p = 0.011) were risk factors for Petersen’s hernia. Longer time interval from emergence visit to laparotomy (p = 0.042) and elevated preoperative procalcitonin (p = 0.033) and C-reactive protein (CRP; p = 0.012) were associated with higher risk of bowel necrosis in Petersen’s hernia. Early surgical intervention resulted in less bowel necrosis rate (p = 0.012) and shorter length of necrotic bowel (p = 0.0041).

**Conclusions:**

Low BMI and distal gastrectomy are independent risk factor for Petersen’s hernia after gastrectomy. Curtailing observing time and executing prompt surgery are associated with bowel viability and better outcome in patients with Petersen’s hernia.

## Background

Gastric cancer is one of the leading malignancies worldwide. Radical gastrectomy is the most effective approach for the management of gastric cancer. Nevertheless, postoperative complications occur frequently after gastrectomy, some of which could be severe and even life-threatening.

Petersen’s hernia was firstly reported by Petersen in 1900 as a rare but severe complication after gastrectomy (1) and is defined as an internal hernia that occurs through the “Petersen space.” Petersen space indicates the defect between Roux limb and transverse colon, which is generated after distal or total gastrectomy. The herniation of either afferent or efferent limb could become strangulated and lead to acute necrosis, leading to severe infection, sepsis, and probably short bowel syndrome. As a consequence, the death rate of Petersen’s hernia could reach 30% (2, 3).

The incidence of Petersen’s hernia is quite low, ranging between 1% and 5% (4, 5). Such low incidence makes it impossible to conduct prospective studies. Most of the current literature is composed of case descriptions or small-size series reports. The clinical characteristics and risk factors for Petersen’s hernia after gastrectomy remain to be elucidated.

In this study, we aimed to collect all Petersen’s hernia cases during longitudinal multiple years and compare them with non-Petersen counterparts within the same period to determine risk factors for Petersen’s hernia. Furthermore, we compared patients with small bowel necrosis to non-necrotic cases in order to find risk factors for bowel necrosis in Petersen’s hernia. Finally, we compared clinical outcome between patients who received early or delayed surgical intervention for the purpose of determining the importance of prompt surgery in Petersen’s hernia.

## Methods

### Patients

Between January 2007 and December 2020, all patients diagnosed with gastric cancer in our hospital were recruited for qualification screening. The inclusion criteria included 1) pathological definitive diagnosis of gastric cancer, 2) radical distal or total gastrectomy, 3) D2 lymphadenectomy, and 4) non-closure of Petersen space during the first gastrectomy. The exclusion criteria were 1) emergent gastrectomy due to acute bleeding or obstruction, 2) transthoracic instead of transabdominal approach, 3) combined resection such as splenectomy or colectomy, 4) remnant gastric cancer, 5) proximal gastrectomy, and 6) Billroth I reconstruction. Petersen defect would not be generated in either proximal gastrectomy with esophagogastric reconstruction or distal gastrectomy with Billroth I reconstruction, which were therefore excluded from this study.

All qualified patients were then divided into two groups. Patients who had Petersen’s hernia confirmed by exploratory laparotomy were entered into Petersen group, and the others were entered into comparative group. Patients in Petersen group were further divided into two subgroups according to the occurrence or absence of bowel necrosis that required surgical resection. Bowel necrosis was determined by surgeons, as the ischemic bowel was not possible to return viable after reduction.

All clinical data including demographics, pathological stage of gastric cancer, type of previous gastrectomy (distal or total), gastrointestinal reconstruction method (Billroth II, Billroth II plus Braun anastomosis, or Roux-en-Y), surgical approach (open or laparoscopic), time interval from onset of hernia-associated symptoms to emergency visit, time interval from emergency visit to exploratory laparotomy, preoperative laboratory data including white blood cell (WBC) count, neutrophil percentage, procalcitonin and C-reactive protein (CRP), postoperative intensive care unit (ICU) stay, hospitalization, complication, and death were collected from the electronic medical records.

### Statistics

All continuous variables were described as mean ± standard error, and all category variables were described as frequency (percentage). For univariate analysis, continuous variables were compared using unpaired Student’s t-test with Welch’s correction where applicable, and categorical variables were compared using chi-square test or Fisher’s exact test where applicable. Cases in Petersen group were paired 1:4 to cases in non-Petersen group using propensity score matching (PSM) to balance potential covariates. All significant variables in univariate analysis were brought into multivariate analysis using binary logistic regression model. Statistical significance was defined as p-value <0.05. All statistical analyses were performed using SPSS statistical package (version 13.0) and GraphPad Prism (version 8.0).

### Ethics

This study was approved by the ethics committee of Nanjing Drum Tower Hospital. As a retrospective study, consent was not required from participants.

## Results

A total of 24 patients with Petersen’s hernia were identified in exploratory laparotomy. The other 1,457 gastric cancer patients during the same period did not develop Petersen’s hernia (non-Petersen group). The overall incidence of Petersen’s hernia after radical gastrectomy was 1.62%.

We first compared the clinical characteristics between patients in Petersen and non-Petersen groups. Univariate analysis identified male predominance (p = 0.037), lower body mass index (BMI) (p < 0.01), and higher proportion of distal gastrectomy (p = 0.022) with statistical significance in Petersen group. Other characteristics including age, reconstruction, approach, and pathological stage were similar between the two groups (**Table 1**).

**Table 1:** Univariate and multivariate analysis for risk factors for Petersen’s hernia.

	Petersen (n = 24)	Non-Petersen (n = 1,457)	p	Multivariate analysis	
				OR (95% CI)	p
Age (years)	66.2 ± 8.9	62.1 ± 11.0	0.069		
Sex (n, %)			**0.037**	3.4 (0.9–21.6)	0.10
Male	22 (91.7%)	1,063 (73.0%)			
Female	2 (8.3%)	394 (27.0%)			
BMI (kg/m^2^)	20.2 ± 1.5	21.8 ± 1.3	**<0.01**	0.3 (0.2–0.5)	**<0.01**
Type of gastrectomy (n, %)			**0.022**	2.5 (1.1–6.3)	**0.041**
Distal	16 (66.7%)	621 (42.6%)			
Total	8 (33.3%)	836 (57.4%)			
Reconstruction (n, %)			0.19		
Billroth II	2 (8.3%)	132 (9.1%)			
Billroth II + Braun	1 (4.2%)	267 (18.3%)			
Roux-en-Y	21 (87.5%)	1,058 (72.6%)			
Approach (n, %)			0.78		
Open	20 (83.3%)	1,232 (84.6%)			
Laparoscopy	4 (16.7%)	225 (15.4%)			
pStage (n, %)			0.83		
I	6 (25.0%)	426 (29.2%)			
II	6 (25.0%)	392 (26.9%)			
III	12 (50.0%)	639 (43.9%)			

BMI, body mass index. Bold values indicate statistical significance.

We next performed multivariate logistic regression analysis to reveal risk factors for Petersen’s hernia. Lower BMI [odds ratio (OR) = 0.3, p < 0.01] and distal gastrectomy (OR = 2.5, p = 0.041) were significantly correlated with higher risk of Petersen’s hernia after gastrectomy in patients with gastric cancer (**Table 1**).

To enhance the power of statistical comparison, we developed two comparative groups using PSM method. Twenty-four cases in Petersen’s group were matched to 96 cases (1:4 ratio) in non-Petersen’s group (**Table 2**). Consistent with pre-matched cohort, male predominance (p = 0.036), lower BMI (p < 0.01), and distal gastrectomy (p < 0.01) showed differences between groups using univariate analysis. Further logistic analysis demonstrated that lower BMI (OR = 0.2, p < 0.01) and distal gastrectomy (OR = 6.2, p = 0.011) were independent risk factors for the development of Petersen’s hernia. Notably, the OR value and p-value became more significant compared to those in pre-matched cohort.

**Table 2:** Propensity score matching for the analysis of risk factors for Petersen’s hernia.

	Petersen (n = 24)	Non-Petersen (n = 96)	p	Multivariate analysis	
				OR (95% CI)	p
Age (years)	66.2 ± 8.9	62.9 ± 8.9	0.11		
Sex (n, %)					
Male	22 (91.7%)	67 (69.8%)	**0.036**	1.0 (0.2–8.1)	0.98
Female	2 (8.3%)	29 (30.2%)			
BMI (kg/m^2^)	20.2 ± 1.5	22.4 ± 1.0	**<0.01**	0.2 (0.1–0.4)	**<0.01**
Type of gastrectomy (n, %)			**<0.01**	6.2 (1.7–28.8)	**0.011**
Distal	16 (66.7%)	34 (35.4%)			
Total	8 (33.3%)	62 (64.6%)			
Reconstruction (n, %)			0.23		
Billroth II	2 (8.3%)	9 (9.4%)			
Billroth II + Braun	1 (4.2%)	17 (17.7%)			
Roux-en-Y	21 (87.5%)	70 (72.9%)			
Approach (n, %)			>0.99		
Open	20 (83.3%)	81 (84.4%)			
Laparoscopy	4 (16.7%)	15 (15.6%)			
pStage (n, %)			0.93		
I	6 (25.0%)	25 (26.1%)			
II	6 (25.0%)	27 (28.1%)			
III	12 (50.0%)	44 (45.8%)			

BMI, body mass index. Bold values indicate statistical significance.

**Figure 1:**
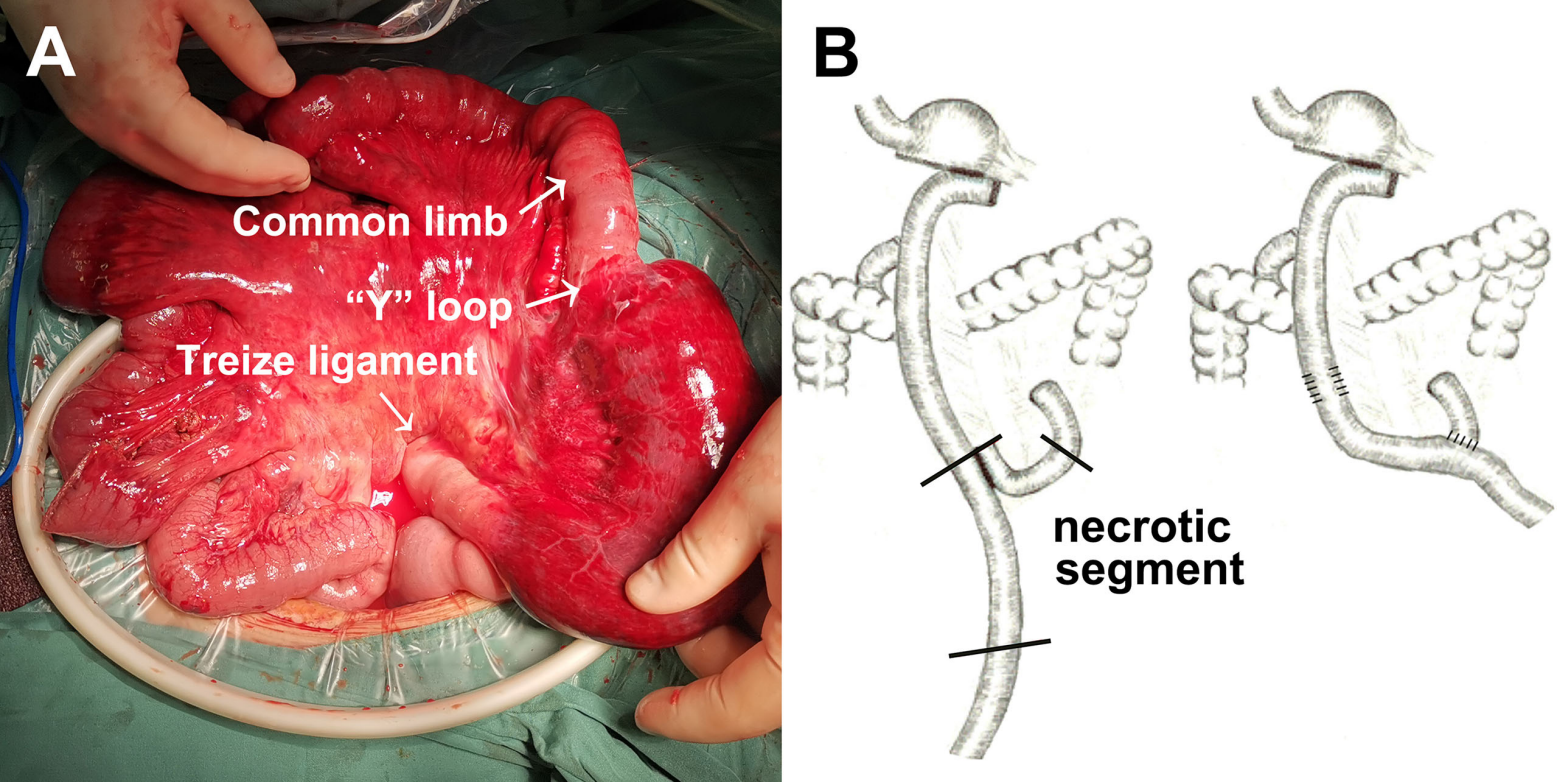
An example of Petersen’s hernia concomitant with small bowel necrosis after gastrectomy. **(A)** Intraoperative detection of small bowel necrosis caused by Petersen’s hernia. **(B)** Graphic illustration of necrotic segment caused by Petersen’s hernia.

Bowel necrosis was found in 13 of 24 cases with Petersen’s hernia (54.2%). **Figure 1** demonstrated a typical case of bowel necrosis caused by Petersen’s hernia. The necrotic segment started from 10 cm distal to Treize ligament and ended until 80 cm distal to jejunojejunal anastomosis (“Y” loop) (**Figure 1A**). The graphic illustration was presented in **Figure 1B**.

We next compared the clinical characteristics between bowel necrosis and non-bowel necrosis groups. Univariate analysis found that the time interval from emergency visit to surgery was significantly longer in necrosis group (p = 0.042). Moreover, patients in bowel necrosis group exhibited significantly higher preoperative procalcitonin (p = 0.033) and CRP (p = 0.012) levels. Consistently, patients with bowel necrosis exhibited longer ICU stay (p = 0.046). There was one case with intra-abdominal infection and surgical site infection in each group. Notably, four patients died in bowel necrosis group, three of whom were found with entire small bowel necrosis and died during laparotomy, and the other case received subtotal resection of small intestine and died from severe infection 2 days after laparotomy. Comparatively, all patients in non-bowel necrosis group survived and were eventually discharged home (**Table 3**).

**Table 3:** Comparison between patients with or without bowel necrosis following Petersen’s hernia.

	Bowel necrosis (n = 13)	Non-bowel necrosis (n = 11)	p	Multivariate analysis	
				OR (95% CI)	p
Age (years)	68.5 ± 5.2	63.5 ± 11.6	0.17		
Sex (n, %)			>0.99		
Male	12 (92.3%)	10 (90.9%)			
Female	1 (7.7%)	1 (9.1%)			
BMI (kg/m^2^)	20.5 ± 1.8	19.7 ± 1.0	0.23		
Concomitant disease (n, %)			0.21		
Yes	3 (23.1%)	6 (54.5%)			
No	10 (76.9%)	5 (45.5%)			
Past surgical history (n, %)			0.65		
Yes	4 (30.8%)	2 (18.2%)			
No	9 (69.2%)	9 (81.8%)			
Previous gastrectomy (m)	33.9 ± 28.8	28.7 ± 28.0	0.66		
Type of gastrectomy (n, %)			0.21		
Distal	7 (53.8%)	9 (81.8%)			
Total	6 (46.2%)	2 (18.2%)			
Reconstruction (n, %)			0.58		
Billroth II	0	2 (18.2%)			
Billroth II + Braun	1 (7.7%)	0			
Roux-en-Y	12 (92.3%)	9 (81.8%)			
Approach (n, %)			0.30		
Open	12 (92.3%)	8 (72.7%)			
Laparoscopy	1 (7.7%)	3 (27.3%)			
pStage (n, %)			0.92		
I	3 (23.1%)	3 (27.3%)			
II	3 (23.1%)	3 (27.3%)			
III	7 (53.8%)	5 (45.4%)			
Time interval from onset to visit (h)	42.8 ± 40.6	46.0 ± 43.6	0.85		
Time interval from visit to surgery (h)	27.7 ± 32.9	7.0 ± 3.2	**0.042**	2.8 (1.1–34.7)	0.19
Preop WBC (×10^9^/L)	11.0 ± 6.8	8.7 ± 3.8	0.33		
Preop neutrophil (%)	82.0 ± 18.7	80.0 ± 10.8	0.75		
Preop procalcitonin (ng/ml)	2.7 ± 3.2	0.7 ± 0.5	**0.033**	2.5 (1.1–10.3)	0.14
Preop CRP (mg/L)	134.2 ± 88.2	53.8 ± 42.1	**0.012**	1.0 (0.9–1.2)	0.27
Postop ICU stay (days)	3.3 ± 3.8	0.7 ± 1.1	**0.046**		
Postop hospitalization (days)	13.3 ± 9.7	8.7 ± 2.3	0.14		
Postop complication (n, %)	1 (7.7%)	1 (9.1%)	>0.99		
Death (n, %)	4 (30.8%)	0	0.098		

BMI, body mass index; WBC, white blood cell; CRP, C-reactive protein; ICU, intensive care unit. Bold values indicate statistical significance.

Subsequent multivariate analysis demonstrated that preoperative procalcitonin and CRP failed to become independent risk factors for bowel necrosis following Petersen’s hernia. Similarly, time interval from visit to surgery was observed to be associated with bowel viability, which however failed to reach statistical difference (**Table 3**).

To further elucidate the association between prompt surgery and bowel viability, we divided patients with Petersen’s hernia into two subgroups, the early and delayed intervention groups according to the time interval from visit to surgery.

The time interval in two groups was 5.8 ± 2.0 and 30.0 ± 31.5 h, respectively (p = 0.022). Preoperative lab results were similar between the groups. Bowel necrosis rate was significantly lower (25.0% vs. 83.3%, p = 0.012) and bowel resection was significantly shorter (20.0 ± 54.4 cm vs. 74.0 ± 94.0 cm, p = 0.0041) in early intervention group. Consistently, patients in early intervention group exhibited shorter ICU stay, shorter hospitalization, less complications, and higher survival rate, although none of these variables reached statistical difference (**Table 4**).

**Table 4:** Comparison between early and delayed surgical intervention of Petersen’s hernia.

	Early intervention (n = 12)	Delayed intervention (n = 12)	p
Time interval from visit to surgery (h)	5.8 ± 2.0	30.0 ± 31.5	**0.022**
Preop WBC (×10^9^/L)	9.6 ± 4.6	10.1 ± 6.5	0.84
Preop neutrophil (%)	82.2 ± 11.1	80.0 ± 18.6	0.73
Preop procalcitonin (ng/ml)	1.4 ± 2.0	2.3 ± 3.1	0.37
Preop CRP (mg/L)	77.0 ± 47.8	112.9 ± 100.1	0.28
Bowel necrosis (n, %)	3 (25.0%)	10 (83.3%)	**0.012**
Bowel resection (cm)	20.0 ± 54.4	74.0 ± 94.0	**0.0041**
Postop ICU stay (days)	0.8 ± 1.1	3.2 ± 3.9	0.083
Postop hospitalization (days)	9.2 ± 2.4	12.8 ± 9.9	0.29
Postop complication (n, %)	0	2 (16.7%)	0.19
Death (n, %)	1 (8.3%)	3 (25.0%)	0.59

WBC, white blood cell; CRP, C-reactive protein; ICU, intensive care unit. Bold values indicate statistical significance.

**Figure 2:**
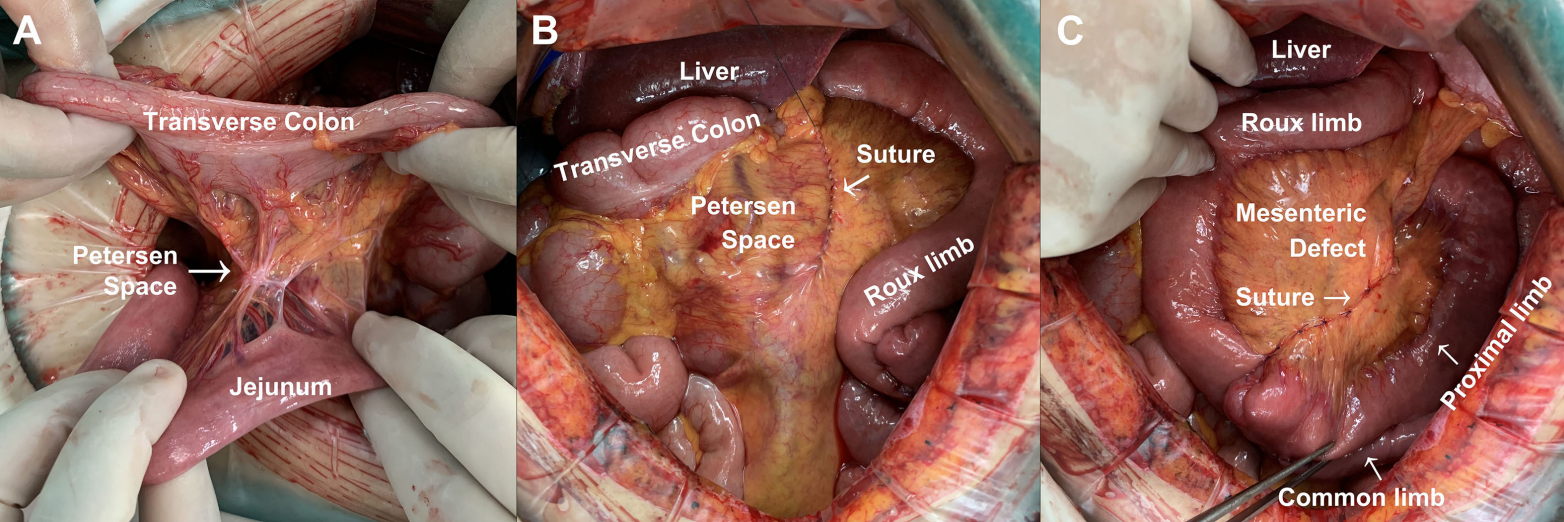
Method for the prevention of Petersen’s hernia during gastrectomy. **(A)** Natural closure of Petersen space. **(B)** Continuous suturing for the closure of Petersen space during gastrectomy. **(C)** Continuous suturing for the closure of mesenteric defect during gastrectomy.

Since January 2021, we have been performing the closure of potential hernia spaces during gastrectomy to prevent postoperative internal hernia (**Figure 2**). In certain patients, there is natural adhesion between transverse colon and proximal jejunum, leading to the absence of Petersen space. Therefore, closure of Petersen space is unnecessary in these patients (**Figure 2A**). In other cases, we conducted a continuous suturing from the root of mesenterium to the margin of intestine for a complete closure of Petersen space (**Figure 2B**). In addition, we conducted continuous suturing to close the mesenteric defect between proximal limb and Roux limb (**Figure 2C**). Since then, none of the patients developed internal hernia in our center.

## Discussion

Gastrectomy is one of the most common procedures in general surgery. Billroth II reconstruction could generate Petersen space, while Roux-en-Y reconstruction could cause both Petersen space and mesenteric defect. Either Petersen space or mesenteric defect causes risk of internal hernia, which results in bowel ischemia, strangulation, and even necrosis (6). If bloodstream at the root of mesenterium is constricted, subtotal even total small intestine needs surgical resection.

There have been a number of articles learning the incidence, clinical manifestation, and outcome of Petersen’s hernia. However, most of them are single-case descriptions or case series reports (7–10). The low incidence of Petersen’s hernia hampers large prospective investigation. Considering the severe consequence of Petersen’s hernia, it is urgent to reveal clinical characteristics and risk factors of Petersen’s hernia.

Herein, our study summarized 24 cases of Petersen’s hernia and compared them with contemporary 1,457 non-Petersen counterparts. By PSM, we found that low BMI and distal gastrectomy are independent risk factors for Petersen’s hernia, which is consistent with a previous study by Han et al. (11). In contrast, Toriumi et al. (12) reported that high BMI is a risk factor for internal hernia after minimally invasive gastrectomy. We assumed that low BMI indicates low weight and less adipose tissue including less visceral fat that is theoretically associated with larger Petersen space. Moreover, distal gastrectomy compared to total gastrectomy results in looser mesenterium of Roux limb, which is therefore associated with larger Petersen space and higher risk of hernia.

Our study investigated risk factors for bowel necrosis in Petersen’s hernia and found that longer waiting time before laparotomy and higher preoperative procalcitonin and CRP are associated with a higher risk of bowel necrosis. Although these parameters failed to reach statistical significance, our data remind surgeons to stay vigilant and perform early intervention. To the best of our knowledge, our study is the first one that identified preoperative waiting time (time interval from visit to surgery) as a risk factor for bowel necrosis in Petersen’s hernia. We also included time interval from initial gastrectomy to onset of hernia and time interval from onset to visit as possible risk factors but concluded that these two parameters were not associated with bowel necrosis in Petersen’s hernia.

To better understand the effect of prompt surgery on bowel viability and postoperative outcome, our study subsequently compared patients receiving early or delayed surgery. We found that early intervention led to less bowel necrosis and shorter bowel resection. This finding, in turn, confirmed the importance of curtailing observing time and executing prompt surgery in patients with suspected Petersen’s hernia. Han et al. (11) also compared surgical outcome between patients in early and late intervention groups and found a slightly lower rate of bowel resection in early group. They, however, neither calculated the length of necrotic bowel nor divided patients into necrotic or non-necrotic groups for risk factor identification (11).

Laparoscopic gastrectomy has been widely adopted. Laparoscopy could reduce surgical adhesion and thereby prevent adhesive bowel obstruction. However, less intra-abdominal adhesion is correlated with increased bowel motility, which brings potential risk of internal hernia (13). Our study found that laparoscopy was not associated with risk of Petersen’s hernia, of which the underlying reasons could include limited sample size and relatively low laparoscopy rate in earlier years. Min et al. (14) discussed the feasibility and advantage of laparoscopic bowel reduction for the treatment of Petersen’s hernia, which brings new insight into the minimally invasive surgery era.

In recent years, a series of studies has demonstrated that closure of Petersen space or mesenteric defect could significantly reduce the risk of internal hernia (13, 15, 16). Therefore, it has been recommended as a routine step during gastrectomy. It remains a controversy to use absorbable or non-absorbable material for suturing. In our center, we use 3-0 non-absorbable suture for the continuous closure of internal defects. Longer follow-up period is necessary to compare the effect of different suture materials and methods for the prevention of internal hernia after gastrectomy.

We are fully aware of our limitations. First, this is a single-center retrospective study, which may bring selection bias. Due to the low incidence of Petersen’s hernia, it is difficult and unpractical to conduct a prospective study. Second, the sample size is limited in the current study. Nevertheless, this has been one of the largest studies in Petersen’s hernia. Future multicenter trials could help recruit more eligible patients for risk factor identification.

## Data Availability Statement

The raw data supporting the conclusions of this article will be made available by the authors without undue reservation.

## Ethics Statement

The studies involving human participants were reviewed and approved by the Ethics Committee of Nanjing Drum Tower Hospital. Written informed consent for participation was not required for this study in accordance with the national legislation and the institutional requirements.

## Author Contributions

SL, QH, and PS collected and analyzed data. LT, SA, JM, FW, XK, XS, FS, and XX performed the surgical interventions. XL, MW, and WG designed and supervised the study. All authors contributed to the article and approved the submitted version.

## Funding

This study was funded by the National Natural Science Foundation of China (82102294 to QH, 81970500 to XS, and 82172645 to WG), the Natural Science Foundation of Jiangsu Province (BK20200052 to SL), and Clinical Trials from the Affiliated Drum Tower Hospital, Medical School of Nanjing University (2021-LCYJ-MS-09 to WG and 2021-LCYJ-PY-17 to SL).

## Conflict of Interest

The authors declare that the research was conducted in the absence of any commercial or financial relationships that could be construed as a potential conflict of interest.

## Publisher’s Note

All claims expressed in this article are solely those of the authors and do not necessarily represent those of their affiliated organizations, or those of the publisher, the editors and the reviewers. Any product that may be evaluated in this article, or claim that may be made by its manufacturer, is not guaranteed or endorsed by the publisher.

## Abbrevations

PH, Petersen’s hernia; WBC, white blood cell; BMI, body mass index; PSM, propensity score matching.
